# Dried blood spots as a matrix for measuring AD biomarkers: optimizing parameters of analysis and evaluating technical stability

**DOI:** 10.1002/alz.093339

**Published:** 2025-01-09

**Authors:** Bruno L. Hammerschlag, Hadia A Fatima, Johanna Celedon, Trevor L Ragas, Hannah A Webster, Steven E Arnold, Pia Kivisäkk

**Affiliations:** ^1^ Massachusetts General Hospital, Boston, MA USA; ^2^ Harvard Medical School, Boston, MA USA; ^3^ Massachusetts General Hospital, Harvard Medical School, Boston, MA USA

## Abstract

**Background:**

An advantage of blood‐based biomarkers of Alzheimer’s disease (AD) is that collection is easily repeatable and minimally invasive. Repeated biomarker measurements provide greater sensitivity for detecting within‐person change, which in clinical trials can allow for reductions in sample sizes required to detect treatment effects and shorten trial periods. We validated Dried Blood Spots (DBS) as a novel matrix for measuring blood biomarkers of AD, as collection is easily repeatable, less burdensome for participants, and more economical than phlebotomy, and shipment and storage are simplified. Our goal was optimizing collection methods and parameters of measurement, and evaluating the technical stability of repeat measurements and day‐to‐day variability in individual study participants.

**Methods:**

We measured levels of phosphorylated tau 217 (pTau217), glial fibrillary acidic protein (GFAP), and neurofilament light (NfL) using commercially available electrochemiluminescent immunoassays in DBS and paired plasma samples. Venous blood was obtained from participants in the Massachusetts Alzheimer’s Disease Research Center’s longitudinal cohort study and patients undergoing diagnostic lumbar punctures at Massachusetts General Hospital. DBS samples were prepared by applying whole blood immediately post‐collection to Whatman 903 filter cards and stored at room temperature. Before analysis, DBS samples were punched out of cards and eluted at room temperature overnight.

**Results:**

We optimized pre‐analytical factors of DBS analysis by investigating differences in elution times and temperatures, and matrix and diluent effects. Using our optimized protocol, GFAP [median (IQR): 4.1 (6.7‐2.2) pg/ml], NfL [29.2 (36.3‐15.2) pg/ml], and pTau217 [4.5 (5.6‐3.3) pg/ml] were detected over the lower limit of quantitation (LLOQ) in all samples. Excellent correlations between plasma and DBS concentrations were observed for GFAP (r=0.90; p<0.0001) and NfL (r=0.96; p<0.0001), while initial validation in a smaller sample size indicated acceptable correlation for pTau217 (r=0.78; p<0.01).

**Conclusions:**

We found measurable levels of three key biomarkers of AD in DBS samples. Concentrations were significantly correlated to levels in paired plasma samples. We are currently evaluating technical repeatability and short‐term variability in capillary DBS levels of pTau217, GFAP, and NfL.

